# Supplementary material for: Neuromorphic computing with multi-memristive synapses
Source: Nat Commun. 2018 Jun 28;9:2514. doi: 10.1038/s41467-018-04933-y (PMC6023896; doi:10.1038/s41467-018-04933-y)
Supplement: Supplementary file 1 — Supplementary Information [file 41467_2018_4933_MOESM1_ESM.pdf]

Supplementary information

Neuromorphic computing with multi-memristive synapses

Boybat *et al.*

SUPPLEMENTARY FIGURE 1: INTER-DEVICE VARIABILITY OF PCM

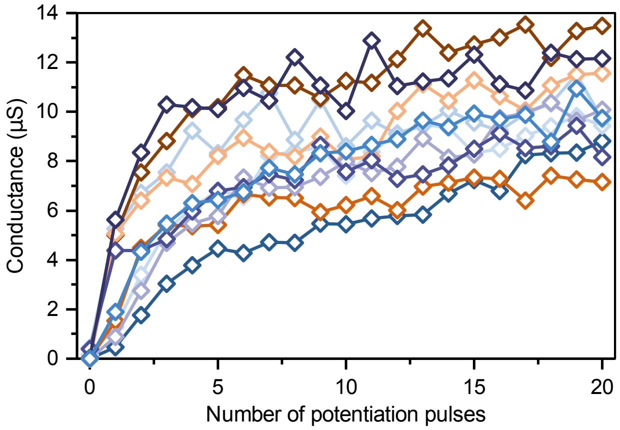

Supplementary Figure 1. **Inter-device variability of PCM.** (a) The evolution of conductance as function of number of potentiation pulses. Programming pulses of 100 µA amplitude and 50 ns width are applied. Data is obtained from 10 PCM devices fabricated in the 90 nm technology node.

SUPPLEMENTARY FIGURE 2: DISTRIBUTION OF NORMALIZED CUMULATIVE CONDUCTANCE CHANGE IN MULTI-MEMRISTIVE SYNAPSES

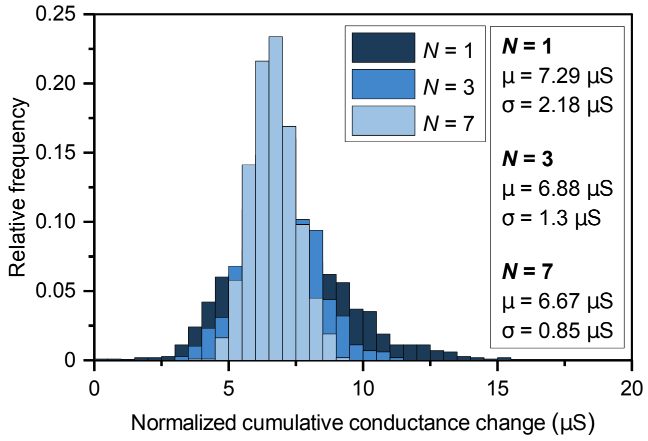

Supplementary Figure 2. **Distribution of normalized cumulative conductance change in multi-memristive synapses.** Distribution of normalized cumulative conductance change after the application of 10, 30 and 70 potentiation pulses to 1, 3, and 7-PCM synapses, respectively. The cumulative conductance change distributions of Fig. 3(b) of the manuscript is normalized by the number of devices N. The means of the distributions are similar, however the standard deviations decrease by $\sqrt{N}$ with increasing number of devices, leading to an overall increase in resolution by $\sqrt{N}$. Potentiation pulses of 100 µA amplitude and 50 ns width are applied. The measurements are based on 1,000 synapses and each device is initialized to a conductance of approximately 5 µS before the application of potentiation pulses. PCM devices are fabricated in the 90 nm technology node.

SUPPLEMENTARY NOTE 1: ILLUSTRATION OF THE ARBITRATION SCHEME WITH SELECTION COUNTER

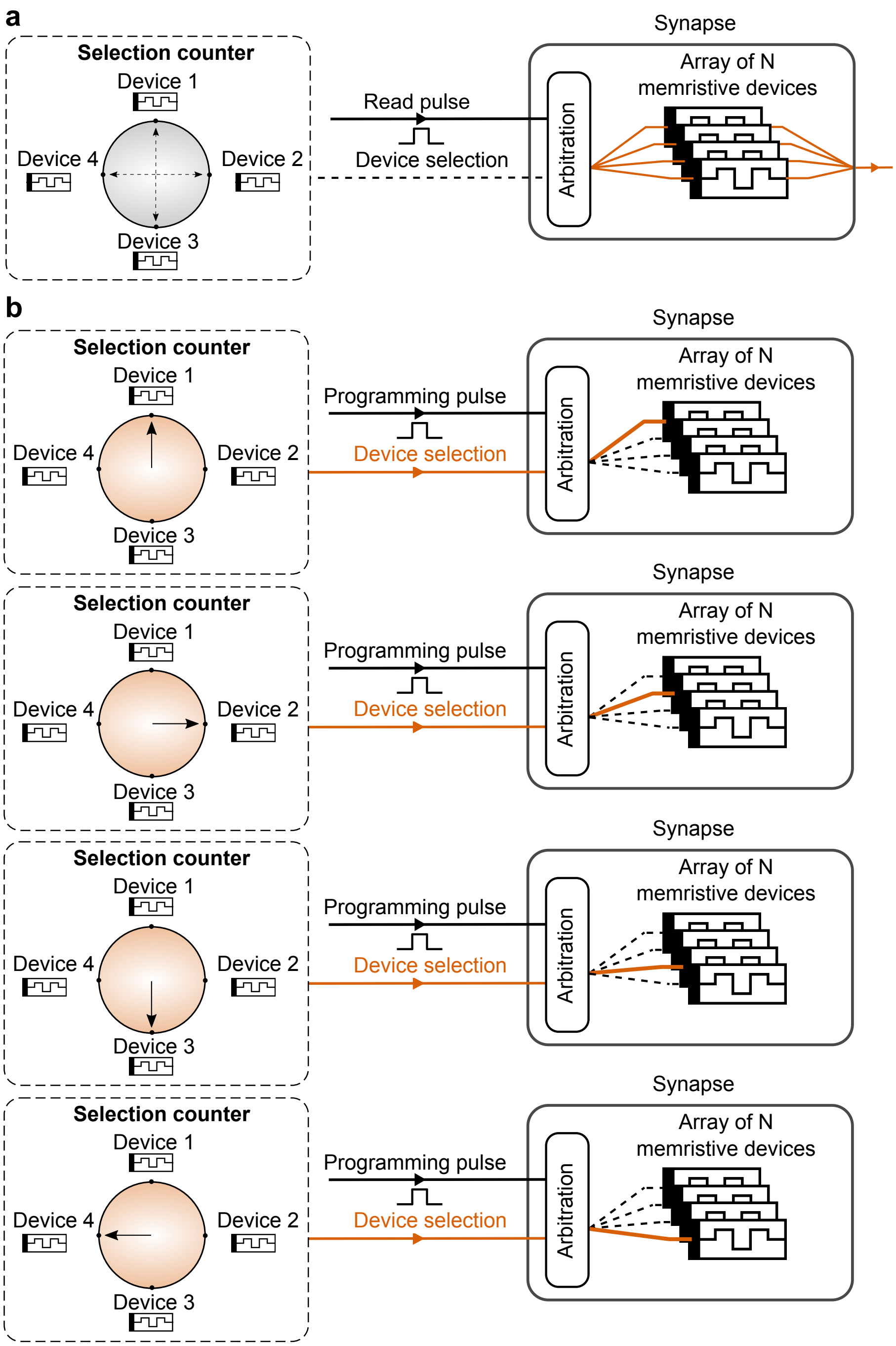

Supplementary Figure 3. **Illustration of the arbitration scheme with selection counter.** Synapse selection by the arbitration module during the implementation of (a) synaptic efficacy and (b) synaptic plasticity is illustrated. The arbitration module receives input from a selection counter only; no potentiation or depression counter is present.

Supplementary Fig. 3 depicts how the conductance value of a multi-memristive synapse is read and programmed with the selection counter. The cartoon shows the device selection in a multi-memristive synapse comprising $N = 4$ memristive devices. The selection counter has a maximum value of 4. Each value the selection counter has corresponds to one of the devices in the multi-memristive synapse. During synaptic efficacy implementation (see Supplementary Fig. 3(a)), the read pulse is applied to all the devices in the synapse, regardless of the value of the selection counter. During synaptic plasticity implementation (see Supplementary Fig. 3(b)), only one of the devices receives the programming pulse. The value of the selection counter is received by the arbitration module and programming pulse is applied to the corresponding device. After the device has been programmed, the value of the selection counter is incremented. This increment rate is 1 in this illustration.

The choice of the increment rate has an impact on the device selection. If the increment rate is set to 2 and assuming that the initial value of the counter is 1, the device selection order would be device number one, three, one, three, and so on and so forth. The counter will never point to devices two and four, because the combination of the counter increment rate and number of devices in the synapse only allows a subset of the devices to be selected. To ensure that all the devices get selected, a mathematical property needs to be satisfied: if the counter increment rate is co-prime with the number of devices, then all the devices will be selected by the counter. Thus, having an increment rate co-prime with the number of devices N guarantees that all devices within each synapse eventually get selected and will receive a comparable number of updates provided there is a sufficiently large number of updates.

SUPPLEMENTARY NOTE 2: ILLUSTRATION OF THE ARBITRATION SCHEME WITH SELECTION AND POTENTIATION COUNTERS

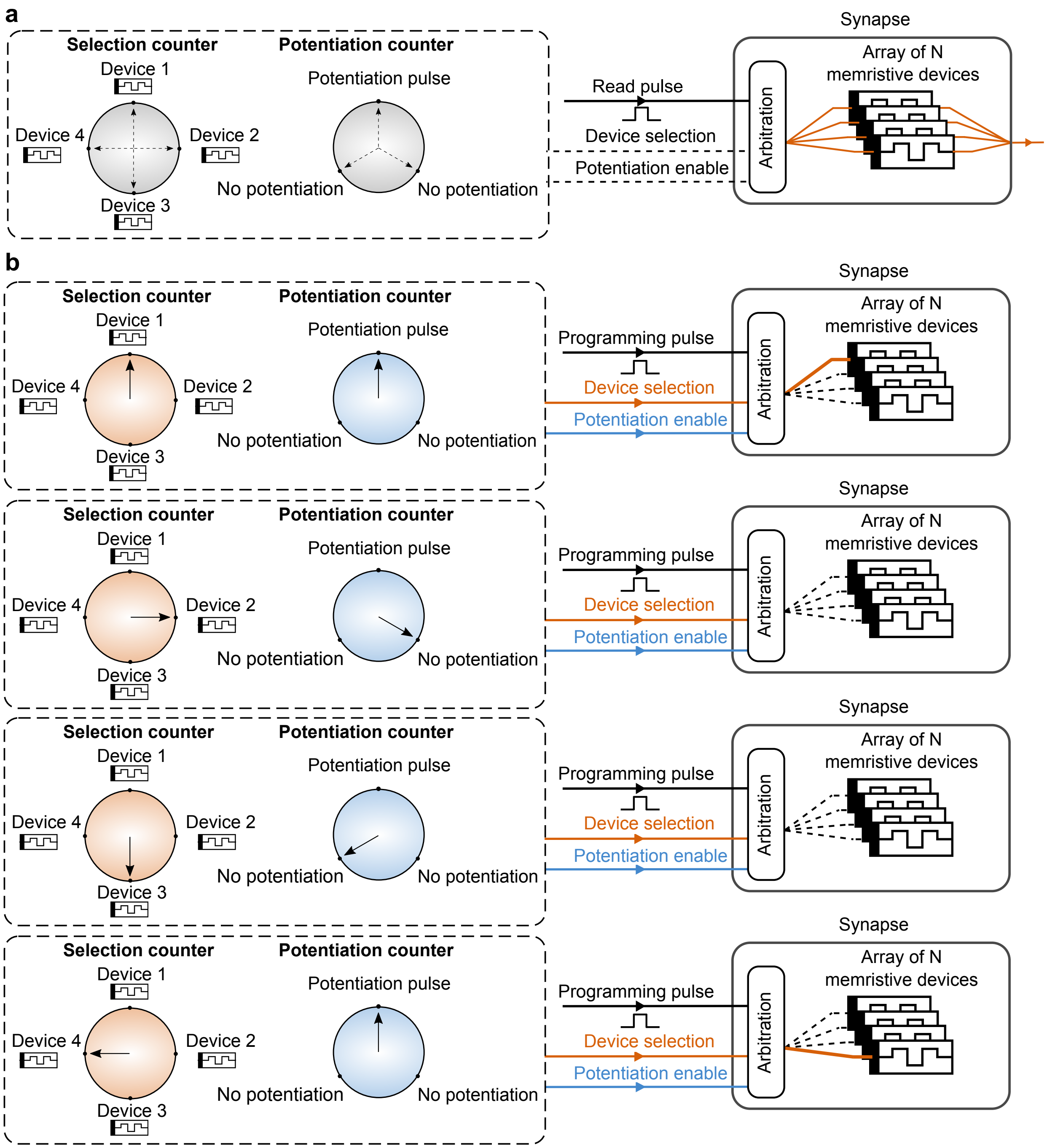

Supplementary Figure 4. **Illustration of the arbitration scheme with selection and potentiation counters.** Synapse selection by the arbitration module during the implementation of (a) synaptic efficacy and (b) synaptic plasticity is illustrated. The arbitration module receives input from a selection counter and a potentiation counter.

Supplementary Fig. 4 builds on the illustration of Supplementary Fig. 3 and shows the synapse selection with the selection and potentiation counters in a multi-memristive synapse. The multi-memristive synapse comprises $N = 4$ memristive devices. The selection counter has a maximum value of 4 and the potentiation counter has a maximum value of 3. A device is selected for potentiation according to the value of the selection counter. The programming pulse is sent to the selected device if the potentiation counter points to the *potentiation pulse*, i.e. if the value of the potentiation counter is 1. If the potentiation counter points to *no potentiation*, i.e. the value of the potentiation counter is other than 1, the pulse is not sent. Thus, the frequency of the potentiation events are adjusted according to the maximum value of the potentiation counter.

The depression counter works in a similar way, enabling or disabling a depression pulse to the device selected by the selection counter.

SUPPLEMENTARY NOTE 3: CROSSBAR-COMPATIBILITY OF MULTI-MEMRISTIVE SYNAPSES

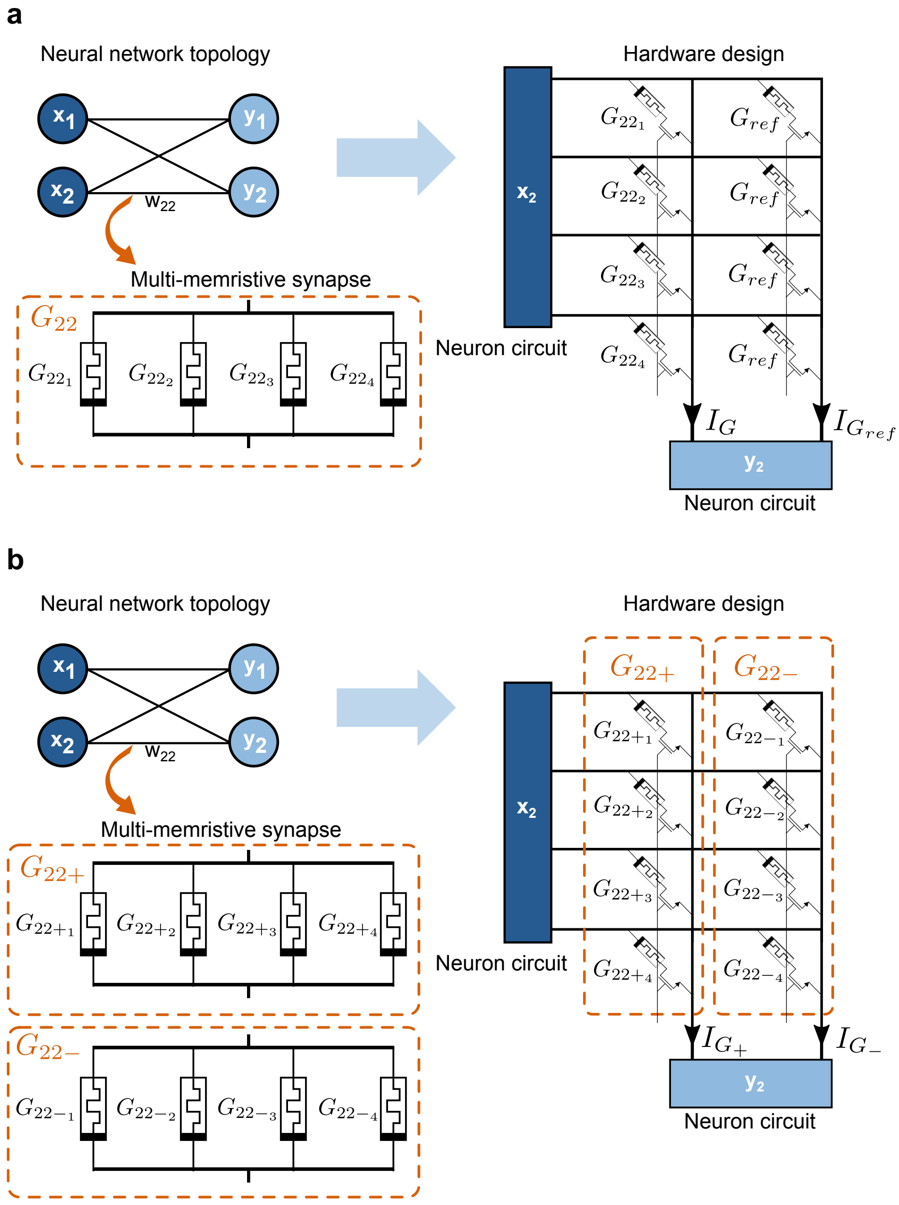

Supplementary Figure 5. **Crossbar-compatibility of multi-memristive synapses.** Crossbar array design for a neural network with multi-memristive synapses (a) with non-differential architecture and (b) with differential architecture.

In a multi-memristive synapse composed of N devices, synaptic efficacy is realized by summing the currents flowing through each of the N devices. For synaptic plasticity, only one device pointed to by the selection counter is programmed. These two operations can be realized in a crossbar array, where N devices are arranged on the same bit line. N word lines, one for each of the N devices, are used and belong to the same input neuron. In a crossbar array with access transistors, the gates of the access transistors can be connected to reduce the number of control signals. For synaptic efficacy, a high voltage is applied to all word lines and the sum of currents is obtained at

the corresponding bit line. For synaptic plasticity, only the word line corresponding to the device to be updated has a high voltage. The device selection is done by the arbitration block, which consists of the selection counter and if applicable the potentiation and depression counters. This arbitration block is common to all neurons and is connected to each of the neuron circuits.

The crossbar array for multi-memristive synapses should be designed to support the desired learning algorithm for neural network training. In a neural network, the synaptic weights can have different ranges. In contrast, the memristive devices have a conductance range between G_{min} and G_{max}, where G_{min}, $G_{\text{max}} > 0$. Let us start with multi-memristive synapses with non-differential architecture. For a learning algorithm where the weight range is limited to either positive or negative real numbers, a scaling factor is sufficient to map the conductance values to weights. This scaling can be done in the output neuron using specialized circuitry (note that the terms input and output neurons refer to the input and output circuitry of the crossbar array, not to the neuron layers of a neural network). However, mapping the device conductance to a weight range spanning both negative and positive real numbers requires adding a bias term. The bias term is proportional to the number of active input neurons, i.e., inputs receiving a high voltage and contributing to the current on the bit line. There are several algorithmic and hardware design options for dealing with the bias term. Firstly, the learning algorithm can be modified such that a weight range spanning both negative and positive real numbers is avoided. Secondly, a communication can be set up between the input neurons and output neuron so that the information on the number of active input neurons is transmitted to the output neurons. Adding a bias term and scaling can then be done at the output neuron. Thirdly, N devices in an additional reference bit line can be used (Supplementary Fig. 5(a)). The conductance of these N devices are pre-initialized to the middle conductance value of a device's conductance range. The overall conductance is calculated in the output neuron as $G - (N \times G_{\text{ref}})$. The same reference bit line can be connected to multiple output neurons; the current on the reference bit line can be reproduced with a current mirror for each of the output neurons. Also, as the conductance of the devices on the reference line is fixed, no additional programming is necessary for them. Let us continue with the differential architecture. Two sets of devices, one representing G_{+} and the other G_{-}, are placed on different bit lines of the crossbar array (Supplementary Figure 5(b)). The overall conductance is calculated as $G_{+} - G_{-}$ in the output neuron. If the weight range required by the algorithm has both positive and negative real numbers, then only a scaling done in the output neuron circuitry can be sufficient. When the required weight range spans either the positive or the negative real numbers, then only one of the bit lines can be used, and the necessary scaling can be done at the output neuron to convert the overall conductance to a weight. The conductance of the devices on the unused bit line can be set to G_{min} so that power dissipation is reduced.

The selection counter is explained to arbitrate between the synapses in a serial manner for simplicity purposes. Using a single global selection counter for arbitration and updating the counter value after each update for the next synapse would increase the training time for networks. However, the proposed arbitration scheme can also be used to program multiple synapses in parallel, in the following way: The same selection counter can be connected to the arbitration block of multiple synapses. In this case, according to the value of the selection counter, devices with the same device number in all synapses will be programmed at the same time. After the devices have been programmed, the selection counter would be incremented. Note that in a crossbar array, only a certain number of devices can be programmed simultaneously due to physical constraints such as the maximum current allowed on a single wire. The proposed scheme can be adapted to such an update scheme.

SUPPLEMENTARY NOTE 4: IMPACT OF DRIFT VARIABILITY ON CONDUCTANCE CHANGE OF PCM

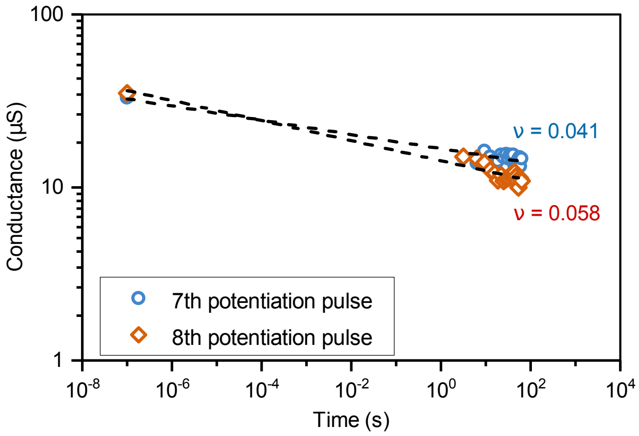

Supplementary Figure 6. **Impact of drift variability on conductance change of PCM.** The temporal evolution of the conductance of a PCM device is shown. To obtain the data, first a depression pulse of amplitude 440 μA was applied to the device. Next, 8 consecutive potentiation pulses of amplitude 110 μA were applied. After the 7th and 8th pulse, the device conductance was measured 20 times consecutively. The initial read measurement was obtained 100 ns after the application of the potentiation pulses, and the 20 read measurements are obtained approximately 3 s apart. The read measurements were used to estimate the drift coefficient. The initial conductance value after the 8th potentiation pulse is higher than the 7th potentiation pulse, however after approximately 60 s, the conductance value after the 8th potentiation pulse is lower than the 7th potentiation pulse. This leads to a perceived negative conductance change after the application of a potentiation pulse.

It is expected that the application of a potentiation pulse results in an increase in the device conductance. However, as shown in in Fig. 2(e) of the manuscript, there are some occurrences of a negative conductance change upon application of a potentiation pulse. A possible explanation for this behavior is the drift variability of PCM. Drift is caused by the structural relaxation of the highly stressed amorphous phase in PCM[1]. At constant temperature, the conductance of these devices exhibit a characteristic $G(t) = G(t_0)\,(t/t_0)^{-\nu}$ relationship, where $G(t)$ denotes the conductance at time t, $G(t_0)$ denotes the initial conductance at time t_0, and ν denotes the drift coefficient. In our devices, we observed a ν value of approximately 0.05. However, there is slight variability associated with the drift coefficient[2]. We suspect that this variability in drift coefficient results in the occasional negative conductance change observed upon application of a potentiation pulse.

To study this further, we conducted experiments on 10,000 PCM devices. First, a depression pulse of amplitude 440 μA was sent to all devices. Then, 8 consecutive potentiation pulses of amplitude 110 μA and width 50 ns were applied. After the 7th and 8th potentiation pulses, the devices were read 20 times. The resulting conductance values associated with one of those devices is shown in Supplementary Fig. 6. The 20 read measurements can be used to investigate the temporal evolution of the conductance and to obtain the drift coefficient. It can be seen that the drift coefficient associated with the state created after the application of the 7th potentiation pulse is 0.041, whereas the drift coefficient associated with the state created after the application of the 8th potentiation pulse is 0.058. This significant difference in the value of drift coefficient is responsible for the fact that the conductance value associated with the state created after application of the

7th potentiation pulse is higher than that after application of the 8th potentiation pulse. Even though the conductance value associated with the state created after the application of the 7th potentiation pulse is lower than that created after application of the 8th potentiation pulse at the first read instance, after a certain period in time (e.g. at the 20th read instance), the conductance value associated with the state created after the application of the 7th potentiation pulse is higher than that after the application of the 8th potentiation pulse. This results in a perceived negative conductance change if the read measurements are conducted after a certain period of time after application of the potentiation pulses.

SUPPLEMENTARY NOTE 5: INPUT SPIKE STREAMS FOR CORRELATION DETECTION

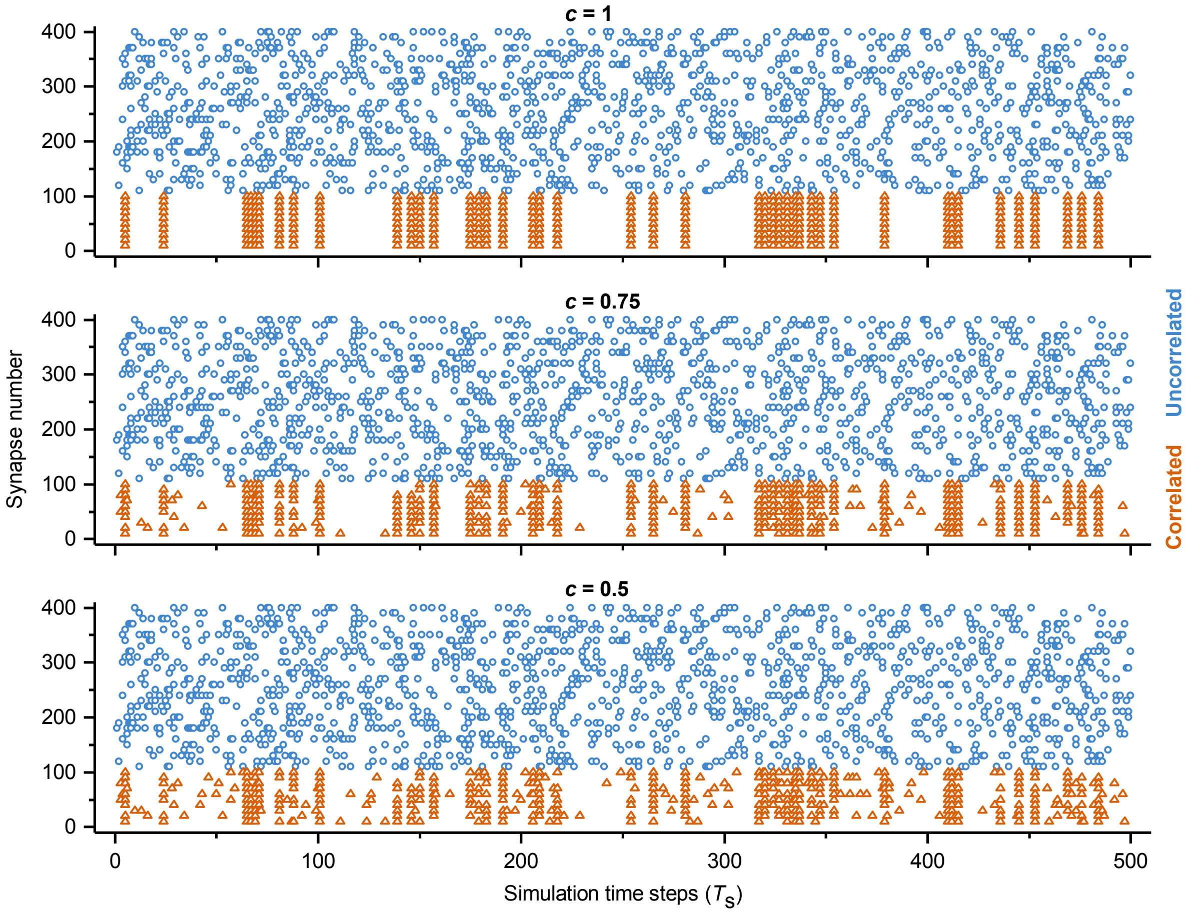

Supplementary Figure 7. **Input spike streams for correlation detection.** Examples of input spike streams for different correlation coefficients c.

Supplementary Fig. 7 shows examples of input spike streams for different correlation coefficients c. In the correlation detection experiment, 100 correlated and 900 uncorrelated spike streams are generated and applied to synapses as presynaptic inputs (see Methods of the manuscript). Correlated spike streams are applied to synapses numbered 1 to 100, and uncorrelated spike streams to synapses numbered 101 to 1,000. For visual simplification, only every 10th of the correlated spike streams is plotted. For the uncorrelated spike streams, every 10th of the first 300 spike streams is shown. As the correlation coefficient c increases, more correlated spikes overlap at the same time instances.

The input spike streams are generated in a similar manner for the large-scale experiment, where 14,400 of the spike streams are correlated and 129,600 of them are uncorrelated. A correlation coefficient c of 0.75 is used for the correlated spike streams.

SUPPLEMENTARY NOTE 6: EXPERIMENTAL DEMONSTRATION OF CORRELATION DETECTION WITH DIFFERENT CORRELATION COEFFICIENTS USING MULTI-MEMRISTIVE SYNAPSES

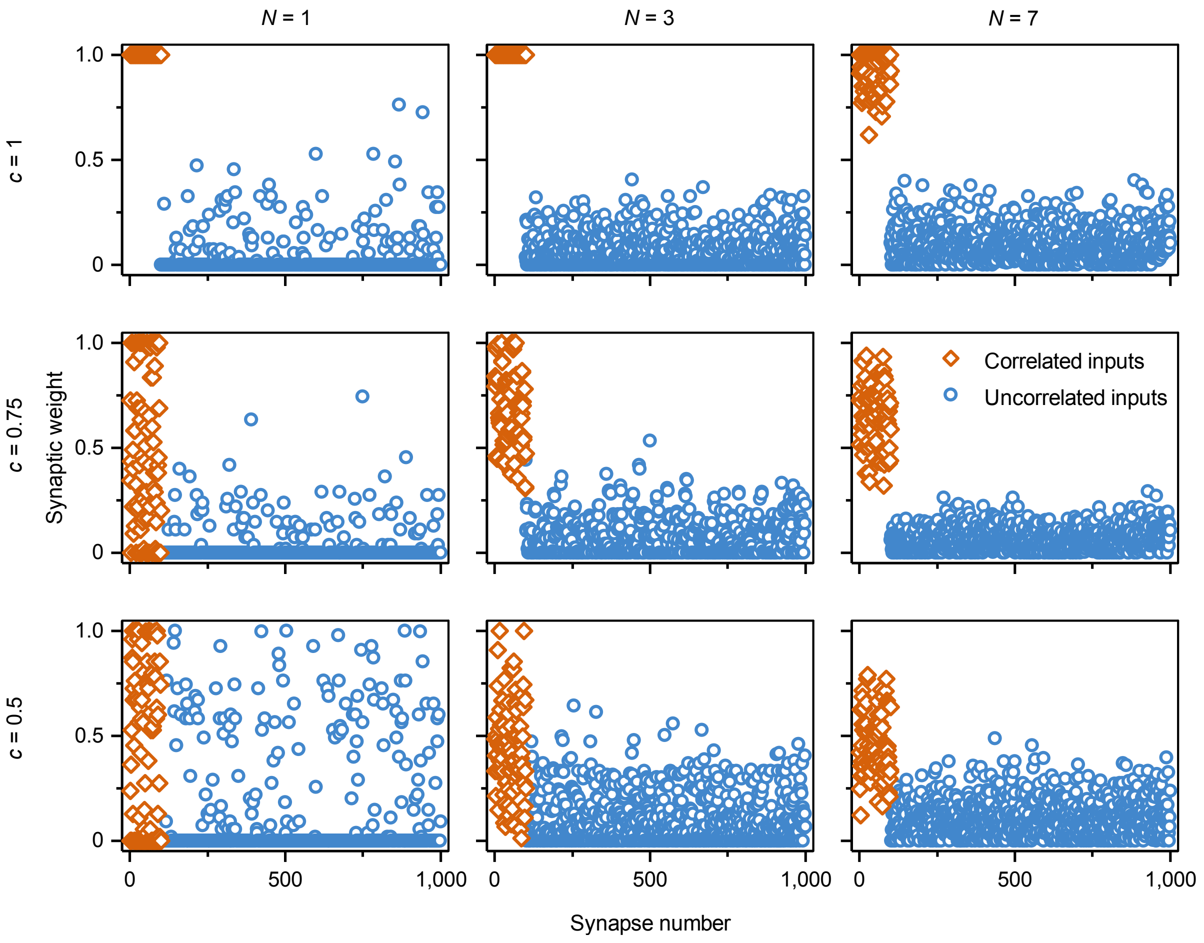

Supplementary Figure 8. **Experimental demonstration of correlation detection with different correlation coefficients using multi-memristive synapses.** Synaptic weights at the end of the correlation detection experiment are shown for different combinations of the correlation coefficient c and the number of PCM devices N in each synapse.

Figure 5(b) of the manuscript shows an experimental demonstration of correlated detection in a spiking neural network network with multi-memristive synapses. The synapses comprise $N = 1$, 3 and 7 devices, and the correlated inputs have a correlation coefficient c of 0.75. In that experiment we picked a threshold weight value that minimizes the number of misclassified inputs to quantify the separation of the synaptic weights for correlated and the uncorrelated inputs. The number of misclassified inputs decreases as the number of devices in a synapses increases. Yet, it can be argued that the number of misclassified inputs reported for $N = 1$ and $c = 0.75$ is low, and a synapse consisting of a single PCM device is sufficient for the task of correlation detection. However, the difficulty of the correlation detection task depends on c (assuming a constant number of correlated inputs)[3]. We conducted experiments in which the correlated inputs have different c in a network with multi-memristive devices. The final synaptic weights are plotted in Supplementary Fig. 8. The number of misclassified inputs for these experiments are listed in Table I and the threshold

weights used to calculate the number of misclassified inputs are listed in Table II. For $c = 1$, even a single memristive device can detect the correlated inputs without any misclassification. However, as c decreases ($c = 0.5$), number of misclassified inputs increases more significantly for a synapse with a single PCM device than for $N = 7$. Multi-memristive synapses proved to be more efficient for correlation detection at lower c than a single PCM device.

	$N = 1$	$N = 3$	$N = 7$
$c = 1$	0	0	0
$c = 0.75$	49	8	0
$c = 0.5$	91	63	36

TABLE I. Minimum number of misclassified inputs (out of 1,000 inputs) for different c and N

	$N = 1$	$N = 3$	$N = 7$
$c = 1$	0.77	0.41	0.41
$c = 0.75$	0.28	0.42	0.30
$c = 0.5$	0.77	0.37	0.32

TABLE II. Thresholds for minimum number of misclassified inputs for different c and N.

SUPPLEMENTARY NOTE 7: WEIGHT-DEPENDENT PLASTICITY ASSOCIATED WITH MULTI-MEMRISTIVE SYNAPSES

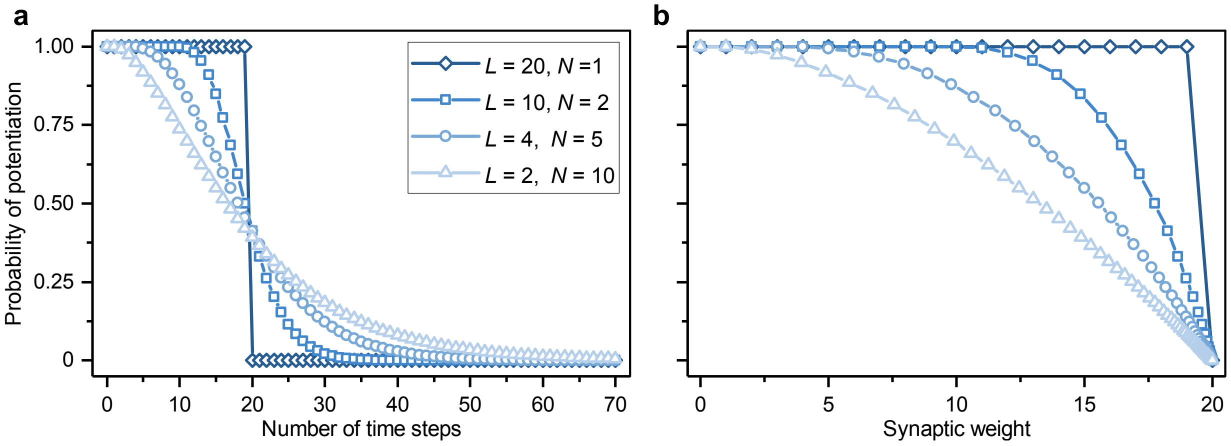

Supplementary Figure 9. **Probability of potentiation in a multi-memristive synapse** (a) as a function of time and (b) as a function of the synaptic weight. L denotes the number of conductance levels of a memristive device and N denotes the number of devices in a multi-memristive synapse.

Because device selection in the multi-memristive synapse is performed based only on the arbitration module, without any knowledge of the conductance values of the individual devices, there is a non-zero probability that a potentiation (depression) pulse will not result in an actual potentiation (depression) of the synapse. This would also translate into a weight-dependent plasticity, whereby the probability to potentiate decreases with increasing synaptic weight and the probability to depress decreases with reducing synaptic weight.

To study this weight-dependent plasticity arising from multi-memristive synapses, we performed a simple analysis, in which such a synapse comprising N memristive devices is created. Each device is assumed to achieve L conductance levels. So the total achievable synaptic weight is $N \times L$. At each time instance t, let $x_i^{(t)} \in [0, 1, \ldots, L]$ denote the conductance level of the i-th device for $i = 1, 2, \ldots, N$.

It is assumed that owing to the counter-based arbitration scheme, devices are selected uniformly at random at a given time step t. Let us also assume that the synapse needs to be potentiated each time it is selected. If the selected device is in an intermediate level, then its conductance level is increased by one. However, if the selected device has reached the maximum conductance value of L (saturation), then it is not possible to increase the conductance level of the device any further and hence it is not possible to potentiate the synapse.

Let Z_t denote the event that potentiation of the synapse occurs at time t. Using the law of conditional probability, the probability of this event can be expressed as follows:

$$\begin{aligned}\Pr(Z_t) &= \sum_{a_1=0}^{L}\sum_{a_2=0}^{L}\ldots,\sum_{a_N=0}^{L} \Pr\left(Z_t, x_1^{(t)} = a_1, x_2^{(t)} = a_2, \ldots, x_N^{(t)} = a_N\right) \\ &= \sum_{a_1=0}^{L}\sum_{a_2=0}^{L}\ldots,\sum_{a_N=0}^{L} \Pr\left(Z_t \middle| x_1^{(t)} = a_1, x_2^{(t)} = a_2, \ldots, x_N^{(t)} = a_N\right) \\ &\times \Pr\left(x_1^{(t)} = a_1, x_2^{(t)} = a_2, \ldots, x_N^{(t)} = a_N\right) \qquad (1)\end{aligned}$$

The conditional probability above is a deterministic function because, conditional on the fixed conductance states of the constituent devices at time t, the probability that a level increase will occur is given by

$$\Pr\left(Z_t \middle| x_1^{(t)} = a_1, x_2^{(t)} = a_2, \ldots, x_N^{(t)} = a_N\right) = \frac{1}{N}\sum_{i=1}^{N} I_L(a_i), \tag{2}$$

where $I_L(a)$ is a function that indicates whether the device is not saturated:

$$I_L(a) = \begin{cases} 1, & \text{if } a < L \\ 0, & \text{if } a = L \end{cases} \tag{3}$$

Thus, it is possible to evaluate probability (1) provided that one can compute the probability of the N devices being in a certain state at time t. There are two distinct types of events that can lead to the devices having a particular state at time t. Firstly, we may have the situation where a non-saturated device i was selected at time $(t-1)$, and $x_i^{(t-1)} = x_i^{(t)} - 1$, thus causing the level to be increased. Alternatively, at the preceding time step, a saturated device may have been selected, thus causing the devices to remain in precisely the same state. Thus, it is possible to compute the state probability recursively, for $t > 0$, as follows:

$$\begin{aligned} &\Pr\left(x_1^{(t)} = a_1, \ldots, x_N^{(t)} = a_N\right) = \\ &\Pr\left(x_1^{(t-1)} = a_1, \ldots, x_N^{(t-1)} = a_N\right) \times \left(1 - \frac{1}{N}\sum_{i=1}^{N} I_L(a_i)\right) \\ &+ \frac{1}{N}\sum_{i=1}^{N} \Pr\left(x_1^{(t-1)} = a_1, \ldots, x_i^{(t-1)} = a_i - 1, \ldots, x_N^{(t-1)} = a_N\right), \end{aligned} \tag{4}$$

where we assume that at the first time step all devices are initialized to zero conductance level:

$$\Pr\left(x_1^{(0)} = a_1, \ldots, x_N^{(0)} = a_N\right) = \begin{cases} 1, & \text{if } a_1 = 0, a_2 = 0, \ldots, a_N = 0 \\ 0, & \text{otherwise} \end{cases} \tag{5}$$

This recursion is complex: $(L+1)^N$ states are required to track all possible device configurations. However, for relatively small L and N, it can be evaluated in a reasonable time. In Supplementary Fig. 9(a), we plot how the probability of potentiation evolves as a function of the time steps for various configurations of L and N. As expected, the probability decreases over time. If a single device is used with 20 levels, we have a step-function behavior. If we use multiple devices with fewer levels, we observe a slower decay. To study how the probability of potentiation depends on the underlying configuration of the devices, we can also compute the average total of the conductance levels at a given time instant t as follows:

$$\hat{W}_t = \sum_{a_1=0}^{L}\sum_{a_2=0}^{L}\cdots\sum_{a_N=0}^{L} \Pr\left(x_1^{(t)} = a_1, x_2^{(t)} = a_2, \ldots, x_N^{(t)} = a_N\right) \times \sum_{i=1}^{N} a_i.$$

In Supplementary Fig. 9(b), we plot the probability of potentiation (at time t) as a function of the average total conductance levels (at time t) for different values of L and N. As expected, if $N = 1$ and $L = 20$, a step function behavior is observed. Unless the single device is saturated, the probability of increase is always exactly 1. Once the device is saturated, the probability becomes zero. For the configurations that use $N > 1$, we observe that the probability of potentiation decays gradually as the devices become more conductive.

This rather simplistic analysis clearly illustrates the weight-dependent plasticity that arises from the use of multi-memristive synapses. This characteristic could have an impact on the overall performance of a neural network. For example, weight-dependent plasticity has been shown to impact the classification accuracy negatively in an artificial neural network[4]. In contrast, weight-dependent plasticity could stabilize a spiking neural network intended to detect temporal correlations[5].

SUPPLEMENTARY NOTE 8: CONDUCTANCE RESPONSE IN A MULTI-MEMRISTIVE SYNAPSE

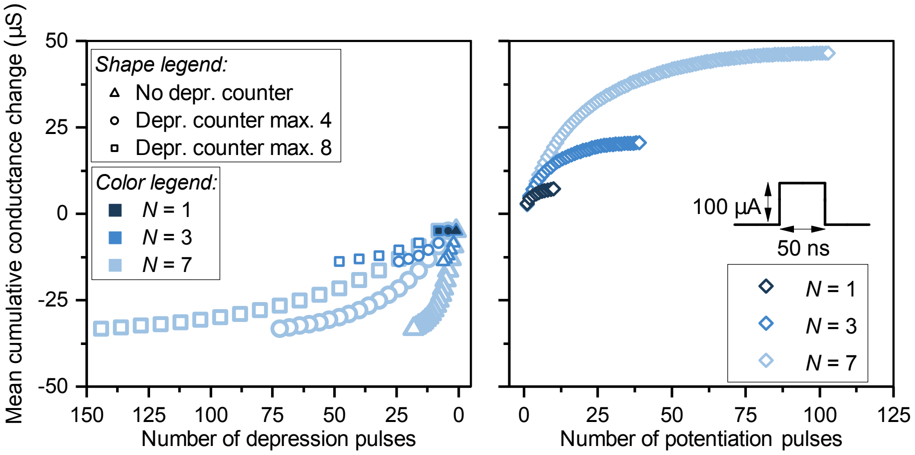

Supplementary Figure 10. **Conductance response in a multi-memristive synapse.** The mean cumulative conductance change is experimentally shown for potentiation and depression for multi-memristive synapses. The synapse comprises $N = 1$, 3 or 7 PCM devices. The selection counter sequence was recorded from an ANN simulation.

Figure 3(a) of the manuscript shows the potentiation and depression behavior of multi-memristive synapses, with the assumption that only one synapse is chosen to be updated repeatedly. Following this assumption, the selection counter selects each device of this synapse one after the other in regular turns. However, this does not fully capture the potentiation and depression behavior of synapses in neural networks. In a spiking neural network, only a random subset of inputs receive spikes, leading to a random subset of synapses getting updates. This implies that the selection of synapses for updates will be done randomly. In addition, at every synaptic weight update, a new device will be picked by the selection counter. In this way, the next device to be updated in a synapse is determined randomly. In contrast, in an artificial neural network (ANN), all synapses might be getting updates at all time steps. In this case, the synapses are selected one after another, creating regular cycles. If the selection counter length is set such that it is co-prime with the total number of synapses in the network, then the selection counter will point to another device of the synapse at each time instance that synapse receives an update.

When devices are selected at random, there are cases where a fully potentiated device receives a potentiation pulse or, inversely, a fully depressed device receives a depression pulse. Both of these events result in an unsuccessful synaptic weight update. Thus, more pulses are required to fully potentiate or fully depress a synapse. This weight-dependent plasticity is analyzed further in Supplementary Note 7.

Supplementary Fig. 10 experimentally demonstrates the mean cumulative conductance change of PCM-based multi-memristive synapses corresponding to the device selection associated with the ANN simulation presented in the main text. Cumulative conductance change measurements are averaged for 1,000 synapses, and each synapse consists of $N = 1$, 3 and 7 PCM devices. The PCM devices were initially programmed to a conductance of approximately 5 µS. Potentiation pulses with a pulse amplitude of 100 µA and a pulse width of 50 ns are used for the measurements. The depression pulse amplitude is 450 µA and width is 50 ns. The selection counter sequence for synapses that are being potentiated and depressed is recorded from an ANN simulation during

training. This ANN has the architecture described in Fig. 4(a) of the manuscript, and the selection counter sequence for 1,000 synapses of the first synapse layer is used. The selection counter length is co-prime with the total number of synapses in the network. In addition, potentiation events are executed before the depression events of that time step in the network. This results in deviations from updating all synapses in a regular order.

SUPPLEMENTARY NOTE 9: ANN AND SNN SIMULATIONS WITH LINEAR DEVICE MODEL

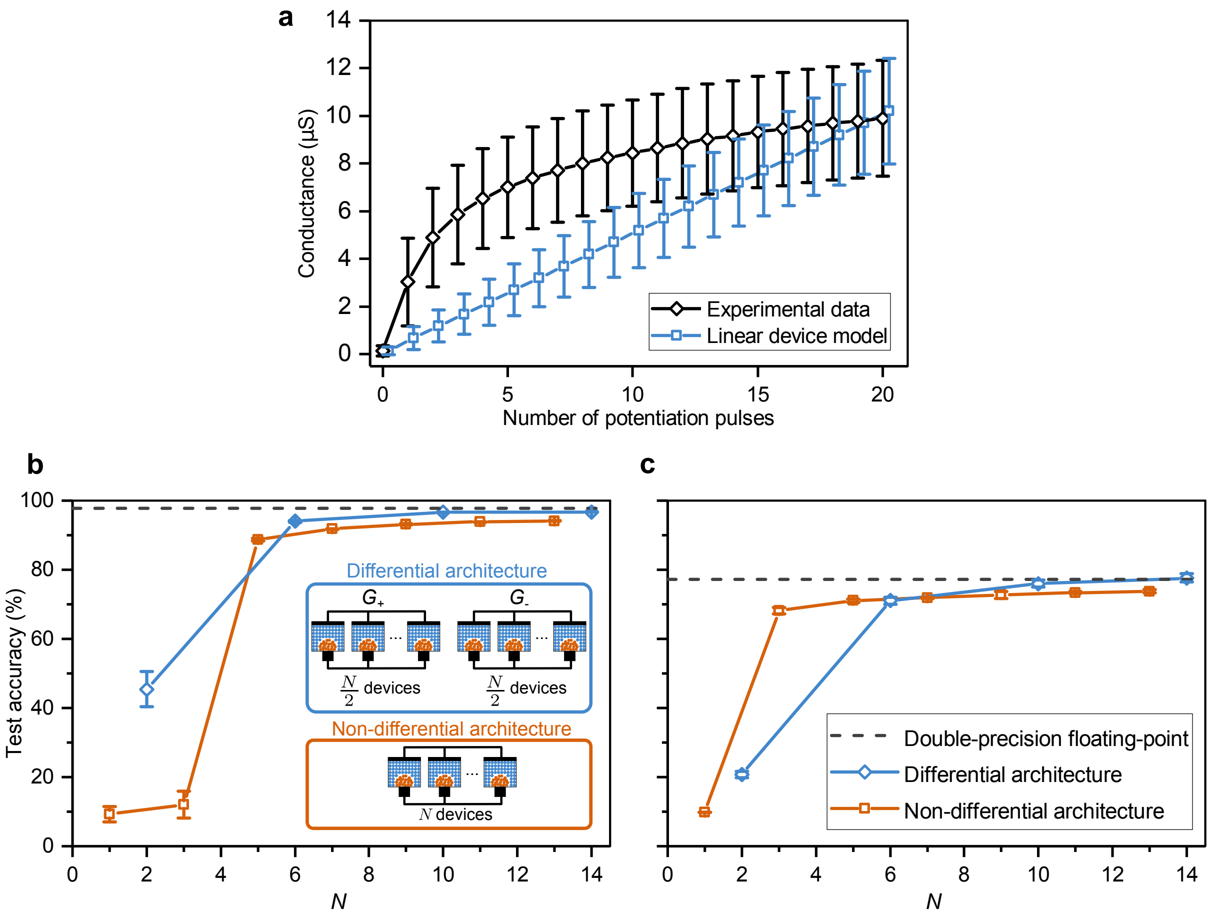

Supplementary Figure 11. **ANN and SNN simulations with linear device model.** (a) Experimental characterization of PCM and the corresponding linear device model. The PCM conductance response is measured with application of pulses of amplitude 100 μA and width 50 ns over 10,000 devices. The devices are fabricated in 90 nm technology node. In the linear device model, conductance change granularity and stochasticity are assumed to be a Gaussian random number with mean and standard deviation equal to 0.5 μS. 10,000 devices are simulated for the model. The error bars represent the standard deviation (1σ). (b) An artificial neural network is trained with backpropagation (c) and a spiking neural network is trained with an STDP-based learning rule to perform MNIST handwritten digit classification. In both (b) and (c), devices in the multi-memristive synapse are simulated using the linear device model. Simulations are repeated for 5 different weight initializations. The error bars represent the standard deviation (1σ).

The ANN and SNN simulations in Fig. 4 of the manuscript are done with a realistic PCM model. The neural network performance is lower than obtained with double-precision floating-point weights. To investigate whether the PCM nonlinearity has an impact on the network performance, we simulated the ANN and SNN using a linear device model (Supplementary Fig. 11(a)). The model parameters are to a large extend based on the experimental PCM behavior (Supplementary Fig. 11(a)), excluding conductance change nonlinearity. In the model, each device is assumed to have a limited dynamic range between 0 μS to 10 μS and covers its dynamic range in

approximately 20 potentation pulses. When applying a potentiation pulse, the conductance change is assumed to be a Gaussian random number with mean and standard deviation equal to 0.5 μS. The device conductance is not increased further if it exceeds 10 μS to model the saturation behavior. The conductance change associated with two different potentiation pulses are assumed to be independent. When applying a single depression pulse, the conductance of the device is set to zero in a deterministic manner.

The training of ANN and SNN is done in a similar way as described in the manuscript (see Section I C and Methods). The results suggest that the test accuracy increases in both ANN and SNN when devices of the multi-memristive synapse are simulated with the linear model (Supplementary Fig. 11(b) and (c)). For the ANN, we can achieve test accuracies exceeding 96.7% with the differential architecture and 94% with the non-differential architecture. Similarly for the SNN, we can obtain test accuracies more than 77% with the differential architecture and 73% in the non-differential architecture. The results suggest that device nonlinearity indeed has an effect on the network performance and improvements in the device conductance response will impact neural network training.

As opposed to the the nonlinear device simulations of the manuscript, for the ANN simulations with the linear device model, no potentiation counter is used for the non-differential architecture. In the differential architecture, the weight of each device is initialized randomly with a uniform distribution in the interval $[\frac{0}{N}, \frac{1}{N}]$. In SNN simulations with the linear device model, a potentiation counter of length 2 is used in the non-differential architecture. In the differential architecture, the weight of each device is initialized randomly with a uniform distribution in the interval $[\frac{2}{5N}, \frac{3}{5N}]$.

SUPPLEMENTARY REFERENCES

[1] Sebastian, A., Krebs, D., Le Gallo, M., Pozidis, H. & Eleftheriou, E. A collective relaxation model for resistance drift in phase change memory cells. In *Proc. IEEE International Reliability Physics Symp. (IRPS)*, MY–5 (IEEE, Monterey, CA, USA, 2015).

[2] Pozidis, H. *et al.* A framework for reliability assessment in multilevel phase-change memory. In *Proc. IEEE International Memory Workshop (IMW)*, 1–4 (IEEE, Milan, Italy, 2012).

[3] Sebastian, A. *et al.* Temporal correlation detection using computational phase-change memory. *Nat. Commun.* **8**, 1115 (2017).

[4] Sidler, S. *et al.* Large-scale neural networks implemented with non-volatile memory as the synaptic weight element: Impact of conductance response. In *Proc. Eur. Solid-State Device Research Conf. (ESSDERC)*, 440–443 (IEEE, Lausanne, Switzerland, 2016).

[5] Gütig, R., Aharonov, R., Rotter, S. & Sompolinsky, H. Learning input correlations through nonlinear temporally asymmetric hebbian plasticity. *J. Neurosci.* **23**, 3697–3714 (2003).
